# Starch Degradation and Sucrose Accumulation of Lily Bulbs after Cold Storage

**DOI:** 10.3390/ijms23084366

**Published:** 2022-04-14

**Authors:** Junpeng Yu, Sujuan Xu, Xinyue Liu, Ting Li, Dehua Zhang, Nianjun Teng, Ze Wu

**Affiliations:** 1 Key Laboratory of Landscaping Agriculture, Ministry of Agriculture and Rural Affairs, College of Horticulture, Nanjing Agricultural University, Nanjing 210095, China; junpengyu2022@163.com (J.Y.); 2019204050@njau.edu.cn (S.X.); 2019104105@njau.edu.cn (X.L.); 2019104104@njau.edu.cn (T.L.); 2017804183@njau.edu.cn (D.Z.); njteng@njau.edu.cn (N.T.); 2 Key Laboratory of Biology of Ornamental Plants in East China, National Forestry and Grassland Administration, College of Horticulture, Nanjing Agricultural University, Nanjing 210095, China

**Keywords:** functional lily, sucrose accumulation, transcriptome

## Abstract

Functional lilies are a group of edible lily cultivars with great potential for landscape application. Low-temperature storage can significantly improve their taste, but the knowledge of this process is largely unknown. In this study, we used the functional lilies ‘Fly Shaohua’ and ‘Fly Tiancheng’ as materials. Through physiological observation and transcriptome analysis during the bulbs’ cold storage, it was found that the starch degradation and sucrose accumulation in bulbs contributed to taste improvement. After 60 d of cold storage, the sucrose accumulation was highest and the starch content was lower in the bulbs, suggesting this time-point was optimal for consumption. Accompanying the fluctuation of sucrose content during cold storage, the enzyme activities of sucrose phosphate synthase and sucrose synthase for sucrose synthesis were increased. Transcriptome analysis showed that many differentially expressed genes (DEGs) were involved in the starch and sucrose metabolism pathway, which might promote the conversion of starch to sucrose in bulbs. In addition, the DEGs involved in dormancy and stress response were also determined during cold storage, which might explain the decreased sucrose accumulation with extended storage time over 60 d due to the energy consumption for dormancy release. Taken together, our results indicated sucrose accumulation was a main factor in the taste improvement of lily bulbs after cold storage, which is attributable to the different gene expression of starch and sucrose metabolism pathways in this process.

## 1. Introduction

Lily (*Lilium* spp.) is an important ornamental crop for production of cut and potted flowers and for application as groundcover in landscaping [1,2,3,4]. In addition, lily bulbs are an extremely important food and medicinal material in China. They are not only rich in nutrients such as carbohydrates, amino acids, dietary fibers, and mineral elements [5,6,7], but also have secondary metabolites such as polysaccharides, saponins, flavonoids, and alkaloids [8,9,10,11,12], which have anti-inflammatory, anti-tumor, antioxidant, anti-aging, and anti-hypoglycemic effects [13,14,15,16]. Among them, some transitional lilies, such as *Lilium pumilum* DC. and *Lilium lancifolium* Thunb., can be also eaten and used in the pharmaceutical industry [17]. Even so, many traditional lilies taste bitter and are difficult to apply in landscaping, and ornamental lilies are not edible. Functional lilies are a series of new lily cultivars with ornamental, edible and medicinal values. Their flowers are large and bright, and the bulbs are delicious. The bulbs of the functional lily can be eaten fresh or deep-processed and used for medicinal purposes.

With the development of science and technology, agricultural products are sold in a variety of ways, far more than local harvesting and local sales. They are often accompanied by long-distance transportation or long-term storage for supplying anytime, anywhere [18]. During storage, transportation and sales, the vigorous post-harvest metabolism of plants will cause changes in the quality of plant products [19]. Cold storage is a widely used method for postharvest preservation of fruits and vegetables [5,20,21]. Under long-term low-temperature conditions, the energy level of cells is decreased, which will not only reduce the nutrient consumption of fruit and vegetable products, but also cause chilling injury [5,22,23]. As to lily bulbs, refrigeration improves their flavors compared to unrefrigerated ones, but the underlying mechanism of taste improvement is still unclear, and the best refrigeration time is also unknown.

In this study, we determined the metabolic changes in carbohydrates, SS activity, and SPS activity in lily bulbs stored for different time-lengths at 4 °C, and RNA-sequencing was also carried out for identification of DEGs during cold storage. By means of cytological observation, physiological and transcriptome analysis, we explored the underlying mechanism for the taste improvement and the suitable time of functional lily bulbs for eating after cold storage.

## 2. Results

### 2.1. Starch Content and Starch Granules Are Gradually Reduced in Bulbs during Refrigeration

**Figure 1:**
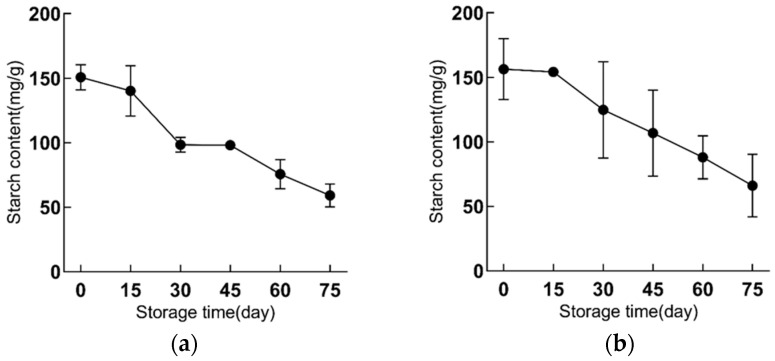
The changes of starch of functional lily during cold storage. (**a**) represents ‘Fly Shaohua’, (**b**) represents ‘Fly Tiancheng’. Starch content has three biological repeats.

With detection of starch content, it was found the starch content of the middle scales in two tested varieties changed similarly under cold storage at 4 °C; that is, the starch content of the scales reduced greatly with the prolongation of storage time (Figure 1). After 75 d of cold storage, the starch content in scales of both cultivars decreased by more than 50%. The trend of a sharp decrease in starch content started from 15 d of cold storage, and from the 0th to the 15th day of storage, the starch content of the two cultivars only slightly decreased (Figure 1a). From the 15th day to the 30th day, the starch content reduced significantly: the content in ‘Fei Tiancheng’ decreased from 154.26 mg/g to 124.84 mg/g (Figure 1b), and the content in ‘Fei Shaohua’ decreased from 140.26 mg/g to 98.37 mg/g (Figure 1a). In ‘Fly Tiancheng’, this sharp downward trend was maintained until the end of cold storage, but the starch content of ‘Fly Shaohua’ showed no significant difference between the 30th day and the 45th day, and after 45 d, the change trend of its starch content was similar to that of ‘Fly Tiancheng’.

**Figure 2:**
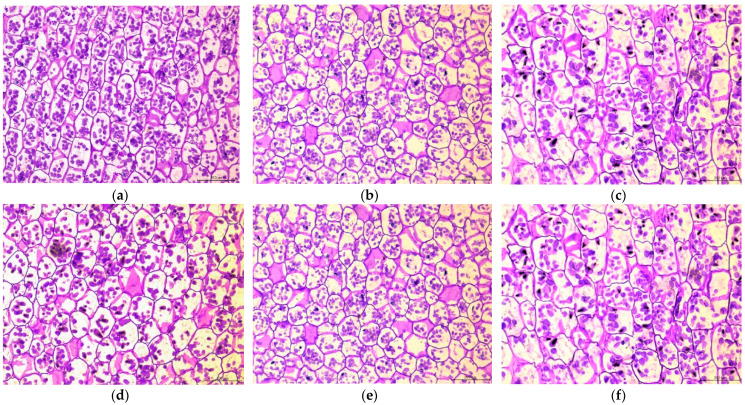
The changes of starch grains in middle scales of functional lily ‘Fly Tiancheng’ during cold storage. (**a**–**f**) represent bulb cells stored at 0 d, 15 d, 30 d, 45 d, 60 d and 90 d of cold storage, respectively. Starch granules are dyed purplish red and nuclei are dyed blue. At least five slices per period were used for observation, and each period showed one representative result.

Through histological observation of ‘Fly Tiancheng’, it was found that the mature starch granules of lily scales were generally oval and spherical, with depressions in the middle. The starch granules exhibited a reduction in size and number during cold storage (Figure 2), resulting in a reduction of starch content. At day 0, abundant starch granules with various sizes in bulb cells could be observed (Figure 2a). At 15 d and 30 d, the starch granules gradually reduced, especially the small ones (Figure 2b,c). It was observed that the edges of some starch granules were blurred, suggesting their degradation (Figure 2c). At 45 d, the size of starch granules was relatively uniform, and the number of starch granules did not decrease significantly compared with 30 d, mainly showing the decreasing size of starch granules (Figure 2d). Compared with 45 d, the number of starch granules was significantly reduced at 60 d, accompanied by less large starch granules, most of them small round or oval starch granules (Figure 2e). There was no significant difference in the number of starch granules at day 75 compared to day 60. But at this time, almost all of them were small starch granules with irregular shapes and blurred edges (Figure 2f). These results indicated that the cold storage activated the degradation of starch in bulbs and caused the decreased starch accumulation.

### 2.2. Sucrose Content Is Increased during Refrigeration

Through UPLC analysis, we found that the extraction of functional lily bulbs did not show obvious chromatographic peaks of glucose and fructose, but a very strong signal of sucrose was observed (Supplementary Figure S1).

**Figure 3:**
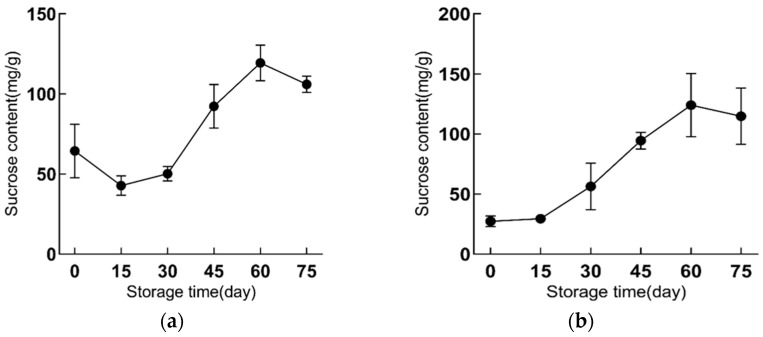
The changes of sucrose of functional lily during cold storage. (**a**) represents ‘Fly Shaohua’, (**b**) represents ‘Fly Tiancheng’. Sucrose content has three biological repeats.

During cold storage of 75 d, the sucrose content in scales of functional lily increased significantly, which increased from the initial 64.36 mg/g to 106.02 mg/g in ‘Fly Shaohua’, and increased from 27.31 mg/g to 114.81 mg/g in ‘Fly Tiancheng’ (Figure 3). The two varieties showed different variation trends of sucrose content in the early cold storage (Figure 3). In the first 15 days of cold storage, the sucrose content of ‘Fly Tiancheng’ increased slightly, while ‘Fly Shaohua’ showed a decline. From the 30th day of refrigeration, the change trend of sucrose content in the scales of ‘Fei Shaohua’ and ‘Fei Tiancheng’ was similar. The sucrose content increased from the 30th to the 60th day and decreased from the 60th to the 75th day of cold storage. The sucrose content increased fastest from the 30th to the 45th day, and the highest sucrose content was on the 60th day. At that time, the sucrose content of ‘Fly Shaohua’ was 119.33 mg/g FW-1 (Figure 3a) and ‘Fly Tiancheng’ was 124.09 mg/g FW-1 (Figure 3b). These results suggested that cold storage accelerated the accumulation of sucrose, and the 60th day might be the appropriate time-point for consumption.

### 2.3. The SPS and SS Activities Are Enhanced during Refrigeration in ‘Fly Tiancheng’

**Figure 4:**
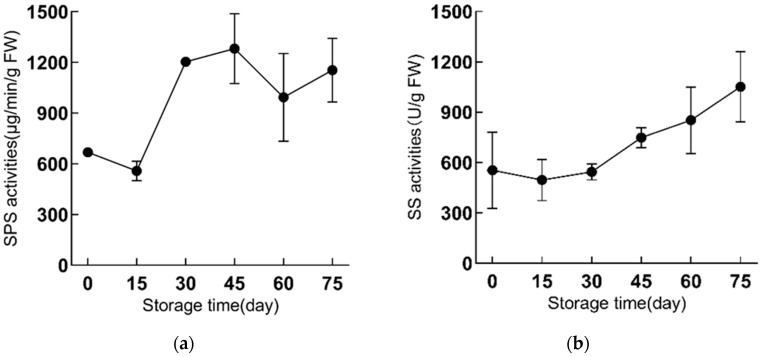
The changes of sucrose of ‘Fly Tiancheng’ during cold storage. (**a**) represents ‘Fly Shaohua’, (**b**) represents ‘Fly Tiancheng’. SPS and SS activity has three biological repeats. SPS, sucrose phosphate synthase (EC 2.4.1.14); SS, sucrose synthase (EC 2.4.1.13).

The SPS and SS are two key enzymes in the starch and sucrose metabolic pathway, and their activity changes are often accompanied by changes of sucrose and starch content. During cold storage at 4 °C, the SPS and SS activities in scales were commonly increased. However, there were large differences in the activity changes of SPS and SS in some stages, and the activity changes of SPS were greater than that of SS during the whole cold storage period (Figure 4a,b). The SS activity was stable from 0 to 30 d with no obvious change (Figure 4b). From the 30th day, the SS activity gradually increased, and this trend continued until the 75th day of refrigeration. Of these, the greatest increase of SS activity occurred from 30 d to 45 d (Figure 4b).

During the first 15 d of cold storage, the SPS activity of the scales decreased slightly, then from 15 d to 30 d, the SPS activity rapidly increased and approximately doubled from 15 d to 30 d (Figure 4a). The SPS activity peaked at 45 d throughout the cold storage period. From the 45th day to the 75th day of refrigeration, the SPS activity fluctuated, but it remained at a high level compared with the initial period of refrigeration (Figure 4a). On the 60th day of refrigeration, the SPS activity decreased to a certain extent compared with the 45th day, which coincided with the time-point when the sucrose content began to decrease. These results indicated both SPS and SS activities are enhanced during refrigeration, which might contribute to the increased sucrose content.

### 2.4. Transcriptome Data Shows Many DEGs Involving Sucrose Metabolism during Cold Storage

A total of 57.15 Gb of transcriptome data with 9 samples including 3 developmental stages—S1 (refrigerated for 0 d), S2 (refrigerated for 15 d), and S3 (refrigerated for 45 d)—was acquired using the DNBSEQ platform. After assembly and de-redundancy, 63,980 unigenes were obtained, and the total length, average length, N50, and GC content were 66,624,749 bp, 1041 bp, 1559 bp, and 46.23%, respectively. Finally, there were 43,687 (NR: 68.28%), 28,188 (NT: 44.06%), 34,474 (SwissProt: 53.88%), 34,897 (KOG: 54.54%), 34,991 (KEGG: 54.69%), 33,251 (GO: 51.97%), and 32,781 (Pfam: 51.24%) unigenes aligned to seven functional databases for annotation. 

**Figure 5:**
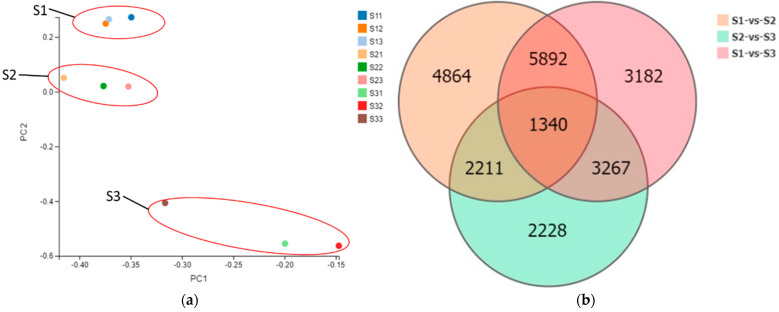

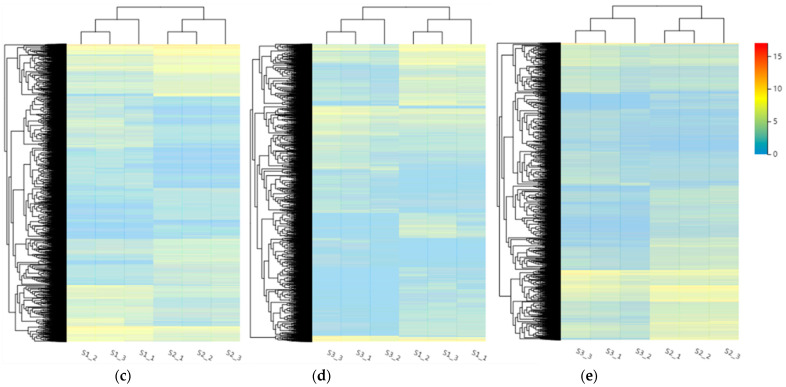
Principal component analysis of different samples, hierarchical cluster, and distribution of DEGs. (**a**) principal component analysis of different samples; (**b**) Venn diagram of DEGs; (**c**–**e**) represent the cluster heat map in S1 vs. S2, S1 vs. S3 and S2 vs. S3, respectively.

From the principal component analysis plot (Figure 5a), it was found that the samples used for transcriptome analysis were relatively consistent within the group, and the differences between groups were obvious. The DEGs were significantly enriched during the different stages of cold storage. There were more DEGs in S1 vs. S2 relative to S1 vs. S3 and S2 vs. S3, suggesting a greater complexity for the regulation of cold storage in S2 (Figure 5b). The hierarchical cluster and distribution of DEGs are shown in Figure 5c–e.

**Figure 6:**
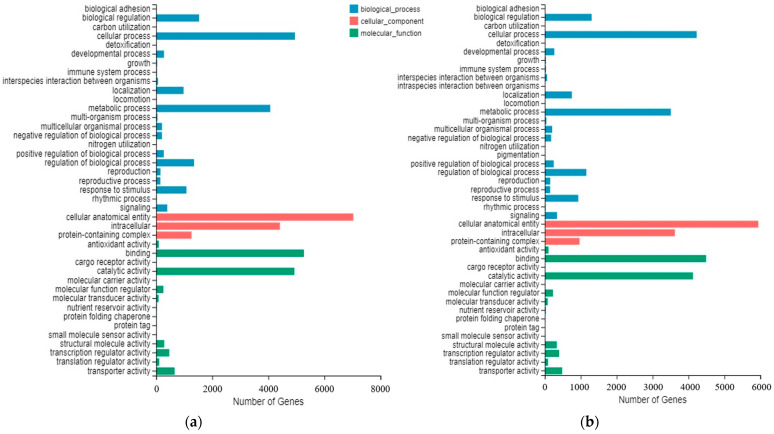

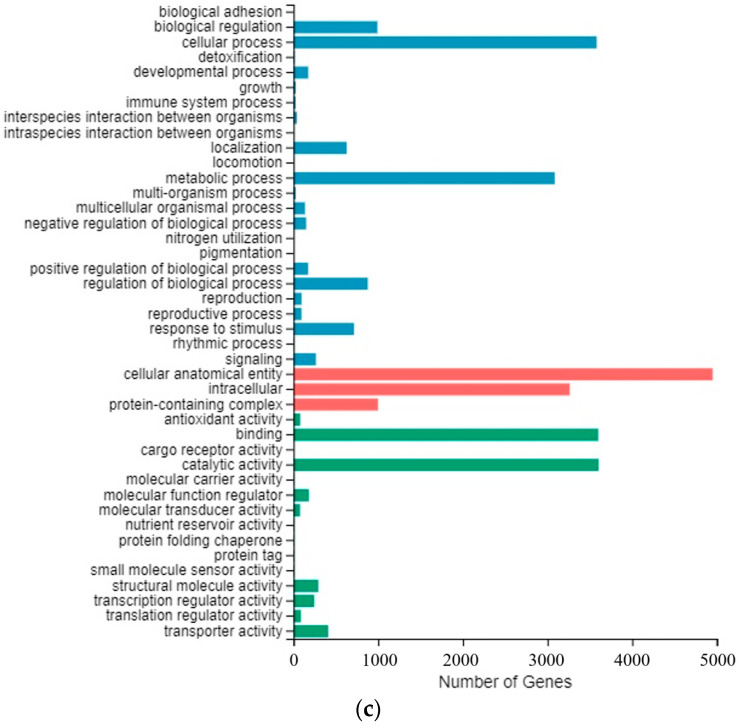
GO enrichment diagrams. (**a**–**c**) represent the secondary GO term annotation for DEGs in S1 vs. S2, S1 vs. S3 and S2 vs. S3, respectively.

The DEGs of the three periods were not significantly different in the annotations in the GO database. In the classification of biological processes, genes annotated as ‘cellular processes and metabolic processes’ account for a large proportion. In the classification of cellular components, there were many genes annotated as ‘cell anatomical entities and intercells’. In the molecular function classification, there were many genes annotated as ‘binding and catalytic activity’ (Figure 6).

**Figure 7:**
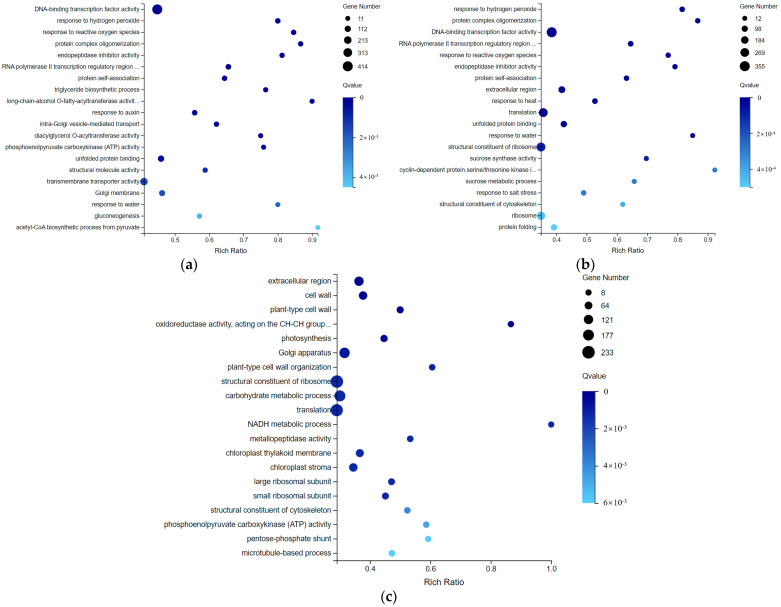
GO pathway enrichment bubble charts. (**a**–**c**) represent the GO pathway name for DEGs in S1 vs. S2, S1 vs. S3 and S2 vs. S3, respectively. The size of the bubble indicates the number of DEGs annotated on a GO term. The color represents the enriched Qvalue value. The darker the color, the smaller the Qvalue.

DEGs in S1 and S2 periods were enriched in GO terms to significantly respond to oxidative stress, transcription, translation, fat synthesis and material transport. In addition, endopeptidase inhibitor activity genes related to protein degradation were also differentially expressed in the two periods (Figure 7a).

Some DEGs in S1 and S3 were also enriched in GO terms to respond to oxidative stress, transcription, and translation related pathways. In addition, DEGs were also significantly enriched in plant response to abiotic stresses such as temperature and water stress, as well as sucrose metabolism and cell cycle related pathways (Figure 7b). S3 is the middle stage of cold storage in this experiment. Compared with the early stage S2 of cold storage, the sucrose metabolism level obviously altered in S3. Compared with the DEGs of S1 vs. S2 and S1 vs. S3, the genes enriched in stress response related pathways were less in S2 vs. S3. The DEGs of S2 and S3 were mostly enriched in plant cell structural substances and pathways related to protein synthesis, carbohydrates, and energy metabolism. In addition, the DEGs were also significantly enriched in the microtubule-based process related to cell division (Figure 7c). Compared with the S3 period, there was only one different factor of cold storage time in S2. Therefore, considering that the differential expression of these genes was related to the accumulation of low temperature for bulb dormancy, these DEGs might be involved in dormancy release.

**Figure 8:**
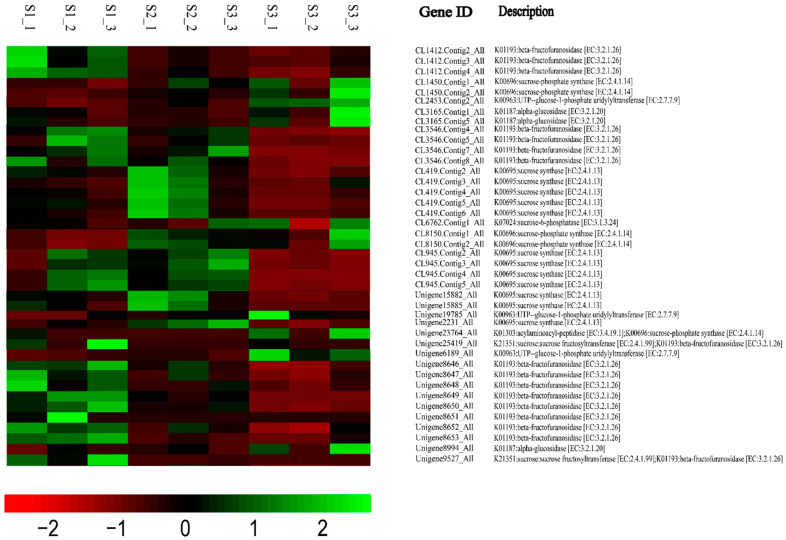
Heatmap of DEGs involved in starch and sucrose metabolism pathway of KEGG. Green indicates up-regulation of genes, red indicates down-regulation, and no change is indicated in black.

**Figure 9:**
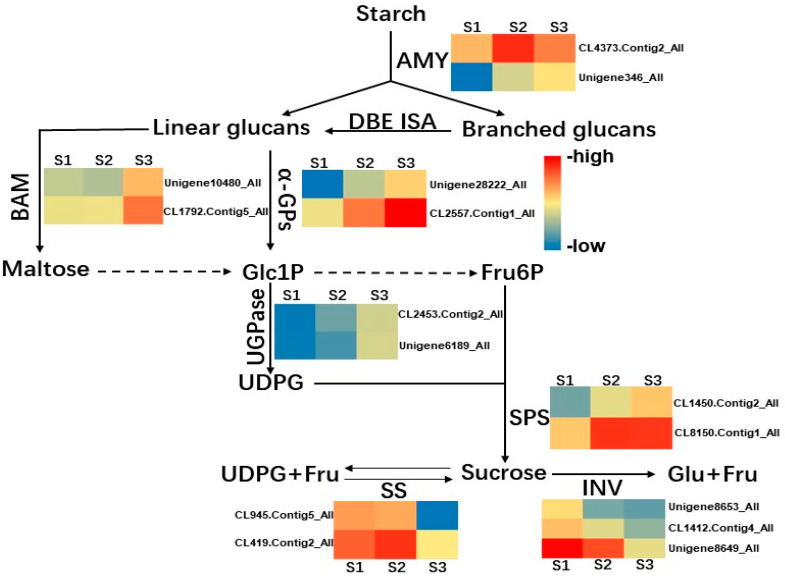
Expression of profiles of unigenes associated with starch and sucrose metabolism. AMY, alpha amylase (EC 3.2.1.1); BAM, beta-amylase (EC 3.2.1.2); α-GPs, α-glucan phosphorylase (EC 2.4.1.1); UGPase, UDP-glucose pyrophosphorylase (EC 2.7.7.9); SPS, sucrose phosphate synthase (EC 2.4.1.14); SS, sucrose synthase (EC 2.4.1.13); INV, invertase (EC 3.2.1.26); Glc1P, glucose 1-phosphate; Fru6P, fructose 6-phosphate; UDPG, UDP-glucose; Fru, fructose; Glu, glucose. Red indicates high expression level and blue indicates low expression level.

The heatmap showed the expression pattern of genes associating with starch and sucrose metabolic pathways, annotated as KEGG (Figure 8). According to the transcriptome data, numerous enzyme genes of starch and sucrose metabolism exhibited highly dynamic changes during cold storage. For example, we analyzed the expression of some important enzyme genes in starch and sucrose metabolic pathways (Figure 9). The expression of six unigenes (AMY: CL4373.Contig2_All, Unigene346_All; BAM: Unigene10480_ALL, CL1792.Contig5_ALL; α-GPs: Unigene28222_All, CL2557.Contig1_All) closely related to starch degradation increased in S2 and S3, which might accelerate starch converted into Glc1P. And then, Glc1P was catalyzed by the accumulated UGPasee (CL2453.Contig2_All, Unigene6189_All) to generate into UDPG, which was an important substrate for sucrose synthesis. In the end, the high content of SPS and SPP catalyzed UDPG to produce sucrose. The expression of transcripts related to the key enzymes SPS (CL1450.Contig2_All, CL8150.Contig1_All) was increased significantly during S2 and S3, which was consistent with the change of the SPS activity. The SS (CL945.Contig5_All, CL419.Contig2_All) is a key enzyme in the starch–sucrose metabolic pathway and has the catalyzing activity for the synthesis and decomposition of sucrose, but it is believed that the main function of SS is to degrade sucrose [24]. According to RNA-sequencing results, the expression level of SS genes was higher in S1 and S2 and showed a low expression level in S3. The expression of INV genes (beta-fructofuranosidase: Unigene8653_All, CL1412.Contig4_All, Unigene8649_All), whose proteins irreversibly catalyze the decomposition of sucrose [25], also showed a downward trend during cold storage. The expression patterns of these genes in the starch and sucrose metabolic pathways explained well the starch degradation and sucrose accumulation during cold storage, confirming that low temperature improved the taste of lily bulbs with more sucrose production.

## 3. Discussion

Because the middle scales of functional lily bulbs are wrapped in the outer scales, there is less water loss during the cold storage process and very little mechanical damage and disease. Moreover, compared with the inner scales, the middle scales have a larger volume and weight. Therefore, the middle scales of functional lily are the best parts for consumption, and it is more meaningful to choose middle scales as experimental materials for investigation.

Previous studies have shown that the lower storage temperature of lily bulbs causes the faster starch–sugar conversion and the higher sugar content after cold storage [26]. However, extreme low temperature would cause chilling injury to the bulbs, which may also affect their flavor. Therefore, in this study, a temperature of 4°C, which has a relatively wide application range in bulbs storage and can ensure a sufficient cold-induced sweetening effect, was used for investigation [27,28,29].

The bulb is an important storage organ of lily. A large amount of starch and soluble sugar is stored in the bulb scales. The scales supply the nutrients and energy for bulb dormancy and bud growth under low-temperature conditions [30] for completing generation. On the other hand, low temperature is also a stress signal which stimulates plant stress responses to avoid damages [31]. The carbohydrate metabolism in bulbs is extremely active under low temperature conditions. Carbohydrates are also important nutrients for humans, and the content and types of food carbohydrates directly affect the quality of food.

The sweetness in plant foods mainly comes from soluble sugar, and the composition of soluble sugar in different plant foods is quite different [32,33,34]. In this study, we found that sucrose was the predominant soluble sugar in bulbs by UPLC analysis. The contents of fructose and glucose are very low and hard to be detected in the UPLC analysis. Fructose and sucrose have high sweetness among soluble sugars, and their contents directly determine the flavor of foods. Due to the low accumulation of fructose in scales, we inferred that the sharp increase in sucrose content during refrigeration is a most important factor that led to the sweeter taste of bulbs.

Starch is derived from photosynthesis of plants. Plants convert light energy into chemical energy and synthesize starch in plastids [35]. Starch not only can serve as an energy substance for us, but also can contribute greatly to the texture properties of foods [36]. The brittleness texture of lotus root is related to the starch content and the ratio of amylose to amylopectin [37]. The studies in buttercup squash show that post-harvest starch-to-sugar conversion not only resulted in an improvement in the flavor of buttercup squash, but also affected its texture [38,39,40]. It is reported that the mealiness of potato varieties is usually associated with high starch content [41]. Our research also verified this rule. The starch content of the middle scales in the two functional lilies reached more than 100 mg/g FW in the early stage of refrigeration. The scales are viscous with pronounced bitterness and less sweetness. However, after being refrigerated for more than 60 days, the texture of scales was crumbly, and the taste of the scales was sweeter. The process of the bulb texture changing from farinose, viscous, and less juicy to brittle, juicy, and sap fluidity is accompanied by the starch degradation during refrigeration. In addition, the starch conversion to soluble sugars in the middle scales may increase osmotic pressure, which promotes the cells of middle scales to absorb water from the outer scales through the basal plate of bulbs, resulting in a crisp texture of the middle scales.

Under the condition of long-term low temperature, the bulb enters the dormancy release process, and a large amount of starch is transformed into sucrose to provide energy and a carbon source for its dormancy break. It was found that sucrose increased gradually during cold storage but decreased slightly after 60 days. According to the previous research results, the dormancy had been released at this time, suggesting that it was consumed as an energy substance in the later stage. Previous studies have shown that the dormancy release of subterranean organs is accompanied by the metabolic processing of sugars, in which ABA and GA pathways play an important role. Transcriptome analysis shows that many genes of ABA and GA pathways are differentially expressed, suggesting that bulbs regulate the dormancy process by affecting hormone response and metabolism and participating in the metabolism of starch and sucrose. Starch can be degraded into glucans and maltose by amylase in plant storage tissues (AMY/BAM) [42], which are transported to the cytoplasm and then catalyzed by UGPase and SPS to produce sucrose. Sucrose can be catalyzed by SS or INV to decompose into hexoses [43,44,45]. As an environmental signal, low temperature can significantly affect gene expression level and material metabolism in plants. Starch is transformed into sucrose, and sucrose as antifreeze can significantly prevent freezing injury [46]. SPS is a key enzyme for the sucrose metabolic pathway, and its activity directly determined the efficiency of sucrose synthesis in plants [47,48,49]. SPS utilizes UDPG as the donor and fructose-6-phosphate as the acceptor to synthesize sucrose-6-phosphate. Sucrose-6-phosphate is dephosphorylated to form sucrose under the catalysis of SPP, and this reaction is almost irreversible; and SPS and SPP form an enzyme complex, so the synthesis of sucrose catalyzed by SPS is also an irreversible reaction. SPS activity is often positively correlated with sucrose accumulation and negatively correlated with starch accumulation in plants [50]. Many studies have shown that the regulation of sucrose metabolism by exogenous stimulus is mainly achieved by affecting the activity of SPS [51,52,53]. Another key enzyme in sucrose metabolism, SS, reversibly catalyzes the formation of fructose and UDPG from sucrose and UDP, but it is generally believed that SS mainly plays a role in sucrose decomposition. SS affects the sink strength of plants and participates in starch metabolism. Overexpression of *SUS4* in potato can increase the content of ADPG and UDPG, and also the starch content [54]. In contrast, silencing SS inhibited starch accumulation in transgenic potatoes [55]. Temperature is the main factor affecting the activity of SPS [56]. In this study, it was observed that the sucrose content in bulbs increased significantly under long-term refrigeration, accompanied by an increase in SPS activity and its expression The decrease of sucrose content was accompanied by the decrease of SS enzyme activity. The synthesis activity of SS increased significantly in S3, but based on RNA-seq data, the expression of SS genes was higher in S1 and S2, and lower in S3 of refrigeration. It was speculated that the increase of sucrose content was also affected by the expression of *SS* genes. When *SS* genes were highly expressed, the number of enzymes increased, and the sucrose decomposition was activated. The expression of *SS* genes was downregulated in S3, which decreased the decomposition of sucrose, and sucrose was accumulated rapidly in the bulbs. The gene expression of several key pathways in the sucrose metabolic network were analyzed by RNA-seq (Figure 9). The unigenes of starch degradation and sucrose synthesis pathways were upregulated during refrigeration, and the unigenes of sucrose degradation pathways were significantly downregulated. Combined with the physiological observation, we verified that the sweeter taste of functional lily was due to the conversion of starch to sucrose during cold storage. The metabolic change of starch and sucrose at low temperature is an extremely complex process, and the enzymes and genes involved in regulation are far more than those presented in this study. The detailed regulation of this metabolic pathway in functional lily bulbs, such as the mechanism of cold signal reception and transduction, activation, and inactivation of related enzymes, and the role of plant hormones in this process need to be further studied in detail.

## 4. Materials and Methods

### 4.1. Plant Materials and Growth Conditions

**Figure 10:**
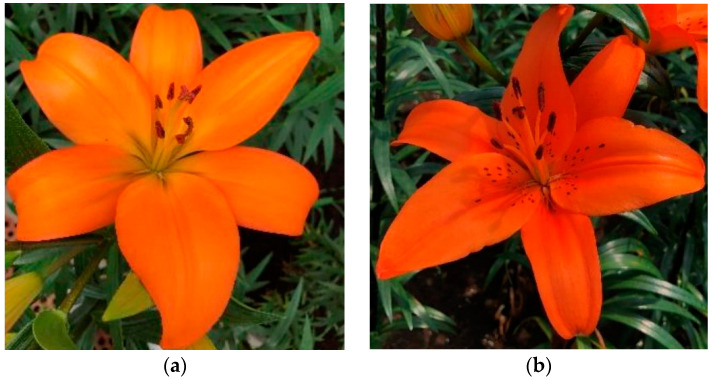

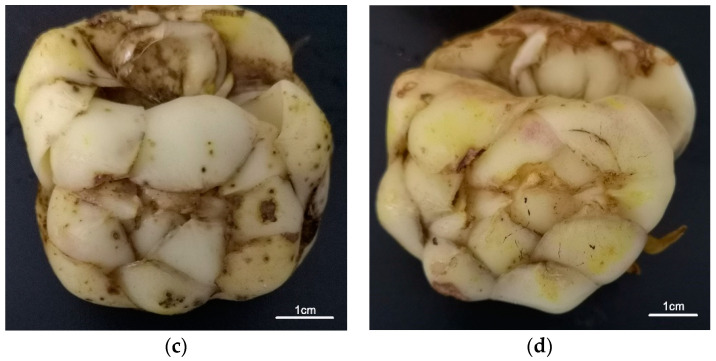
Pictures of functional lilies. (**a**) flower of ‘Fly Tiancheng’ (**b**) flower of ‘Fly Shaohua’ (**c**) bulb of ‘Fly Tiancheng’ (**d**) bulb of ‘Fly Shaohua’.

The bulbs of functional lily ‘Fly Shaohua’ and ‘Fly Tiancheng’ were used in this study, which were preserved in the lily germplasm resources conservation center of Nanjing Agricultural University (Nanjing, China) (Figure 10). The bulbs’ diameters of ‘Fly Tiancheng’ and ‘Fly Shaohua’ used in the experiment were 5–6 cm and 6–7 cm, respectively. The bulbs without diseases, pests, and mechanical damages were harvested in the end of July and stored in a 4 °C refrigerator for cold storage.

At the time-points of 0, 15, 30, 45, 60 and 75 d after low-temperature storage, three bulbs with same size were selected and the middle scales of bulbs were used for investigation.

### 4.2. Detection of Carbohydrate Contents

The 0.1 g tissue was cut from the center of scales with an anatomical knife. After grinding, it was placed in a centrifugal tube, adding 1 mL 80% ethanol, and then centrifuged at 10,000 rpm for 12 min. It was extracted at 40 °C for 1 h. It was centrifuged at 10,000 rpm for 12 min and the supernatant was collected. Then, 1ml of 80% ethanol was added to the filter residue; it was then extracted and centrifuged again, and combined with the supernatant. After steaming dry in a 90 °C water bath, the volume was fixed to 10 mL with 80% ethanol, filtered with 0.22 μm filter membrane, and analyzed with a Waters Ultra Performance Liquid Chromatography UPLC ACQUITY H-Class (Poway, CA, USA) instrument. UPLC conditions: mobile phase: acetonitrile (1% ammonia): water = 85:15, flow rate = 0.2 mL/min, operating column temperature 45 °C, injection time 15 min, injection volume 2 μL; ELSD detector, nitrogen pressure 25 Psi, drift tube temperature 55 °C, sprayer temperature 25 °C. Chromatographic column: UPLC ACQUITY BEH Amide 1.7 μm 2.1 × 100 mm.

The starch content was determined using a plant starch content test kit by anthrone sulfuric acid colorimetry purchased from Nanjing Jiancheng Bioengineering Institute (Nanjing, China).

### 4.3. Histological Observation of Scales

The middle scales of bulbs at different storage stages were sampled and fixed in FAA solution. Histological sections were performed with the conventional paraffin section method, and the samples were cross-sectioned with a thickness of 12 μm. The sections were stained with Periodic acid–Schiff stain and observed under a Leica DM6B microscope (Wetzlar, Germany).

### 4.4. Analysis of Sucrose Phosphate Synthase and Sucrose Synthase Activity

The SPS activity was determined using a sucrose phosphate synthase kit purchased from Suzhou Keming Biotechnology Co., Ltd. (Suzhou, China). The determination principle is that the reaction product sucrose phosphate reacting with resorcinol shows color change, and there is a characteristic absorption peak at the wavelength of 480 nm. The activity in the synthetic direction of SS was determined using a sucrose synthase activity detection kit purchased from Solarbio Biotechnology Co., Ltd. (Beijing, China). The determination principle is that the reaction product sucrose reacting with resorcinol shows color change, and there is a characteristic absorption peak at the wavelength of 480 nm.

### 4.5. Total RNA Extraction

The ethanol precipitation protocol and CTAB-PBIOZOL reagent were used for the purification of total RNA from the bulbs according to the manual instructions. The tissue samples, about 80 mg, were ground with liquid nitrogen into powder, and then the powder samples were transferred in 1.5 mL of preheated 65 °C CTAB-pBIOZOL reagents. The samples were incubated by a mixer for 15 min at 65 °C to permit the complete dissociation of nucleoprotein complexes. After being centrifuged at 12,000× *g* for 5 min at 4 °C, the supernatant was added, 400 μL of chloroform per 1.5 mL of CTAB-pBIOZOL reagent, and centrifuged at 12,000× *g* for 10 min at 4 °C. The supernatant was transferred to a new 2.0 mL tube that added 700 μL acidic phenol and 200 μL chloroform, followed by centrifuging 12,000× *g* for 10 min at 4 °C. The aqueous phase was added, an equal amount of chloroform, and centrifuged at 12,000× *g* for 10 min at 4 °C. The supernatant was added, an equal amount of isopropyl alcohol, and placed at −20 °C for 2 h for precipitation. After that, the mix was centrifuged at 12,000× *g* for 20 min at 4 °C, and then the supernatant was removed. After being washed with 1 mL of 75% ethanol, the RNA pellet was air-dried in the biosafety cabinet and was dissolved by adding 50 µL of DEPC-treated water. Subsequently, total RNA was qualified and quantified using a Nano Drop and Agilent 2100 bioanalyzer (Thermo Fisher Scientific, Waltham, MA, USA).

### 4.6. Construction of mRNA Library

Oligo (dT)-attached magnetic beads were used to purify mRNA. Purified mRNA was fragmented into small pieces with a fragment buffer at an appropriate temperature. Then, first-strand cDNA was generated using random hexamer-primed reverse transcription, followed by a second-strand cDNA synthesis. Afterwards, a Tailing Mix and RNA Index Adapters were added by incubating to end repair. The cDNA fragments obtained from the previous step were amplified by PCR, and products were purified by Ampure XP Beads, then dissolved in EB solution. The product was validated on the Agilent Technologies 2100 bioanalyzer for quality control. The double-stranded PCR products from the previous step were heated, denatured, and circularized by the splint oligo sequence to get the final library. The single strand circle DNA (ssCir DNA) was formatted as the final library. The final library was amplified with phi29 to make a DNA nanoball (DNB), which had more than 300 copies of one molecule, DNBs were loaded into the patterned nanoarray, and single-end 50 bases reads were generated on BGIseq500 platform (BGI, Shenzhen, China).

### 4.7. De Novo Assembly and Gene Functional Annotation

This project used the filtering software SOAPnuke independently developed by BGI (Shenzhen China) for filtering. The clean reads were aligned to the reference gene sequences using Bowtie 2 (College Park, MD, USA), and then the expression levels of genes and transcripts were calculated using RSEM (Madison, WI, USA) [57,58]. Sequences were assembled with Trinity (Jerusalem, Israel) to construct a sequencing library. The DEseq2 method is based on the principle of negative binomial distribution. Based on the theory of negative binomial distribution, this project used DEseq2 (Heidelberg, Germany) to detect DEGs [59,60]. According to the GO and KEGG annotation results and official classification, we classified the differential genes and used the Phyper function in R software (Auckland, New Zealand) for enrichment analysis. The data obtained by sequencing were analyzed through the interactive reporting system (BGI-Shenzhen, China). All the downstream analyses were based on clean, high-quality data.

## 5. Conclusions

Through physiological observation and transcriptome analysis during the bulbs’ cold storage, we found the expression of genes related to starch degradation and sucrose synthesis was increased, and the expression of genes related to sucrose degradation was decreased, which might have caused the starch degradation and sucrose accumulation in the bulbs. The starch degradation and sucrose accumulation contributed to the improvement of the edible quality of functional lily bulbs. After 60 d of cold storage, the sucrose accumulation was highest and the starch content was lower in bulbs, suggesting this time-point is optimal for consumption.

## Data Availability

Not applicable.

## Supplementary Materials

The following supporting information can be downloaded at: https://www.mdpi.com/article/10.3390/ijms23084366/s1.
