# Supplementary material for: Starch Degradation and Sucrose Accumulation of Lily Bulbs after Cold Storage
Source: Int J Mol Sci. 2022 Apr 14;23(8):4366. doi: 10.3390/ijms23084366 (PMC9029042; doi:10.3390/ijms23084366)
Supplement: Supplementary file 1 [file ijms-23-04366-s001.zip › ijms-1678689-supplementary.pdf]

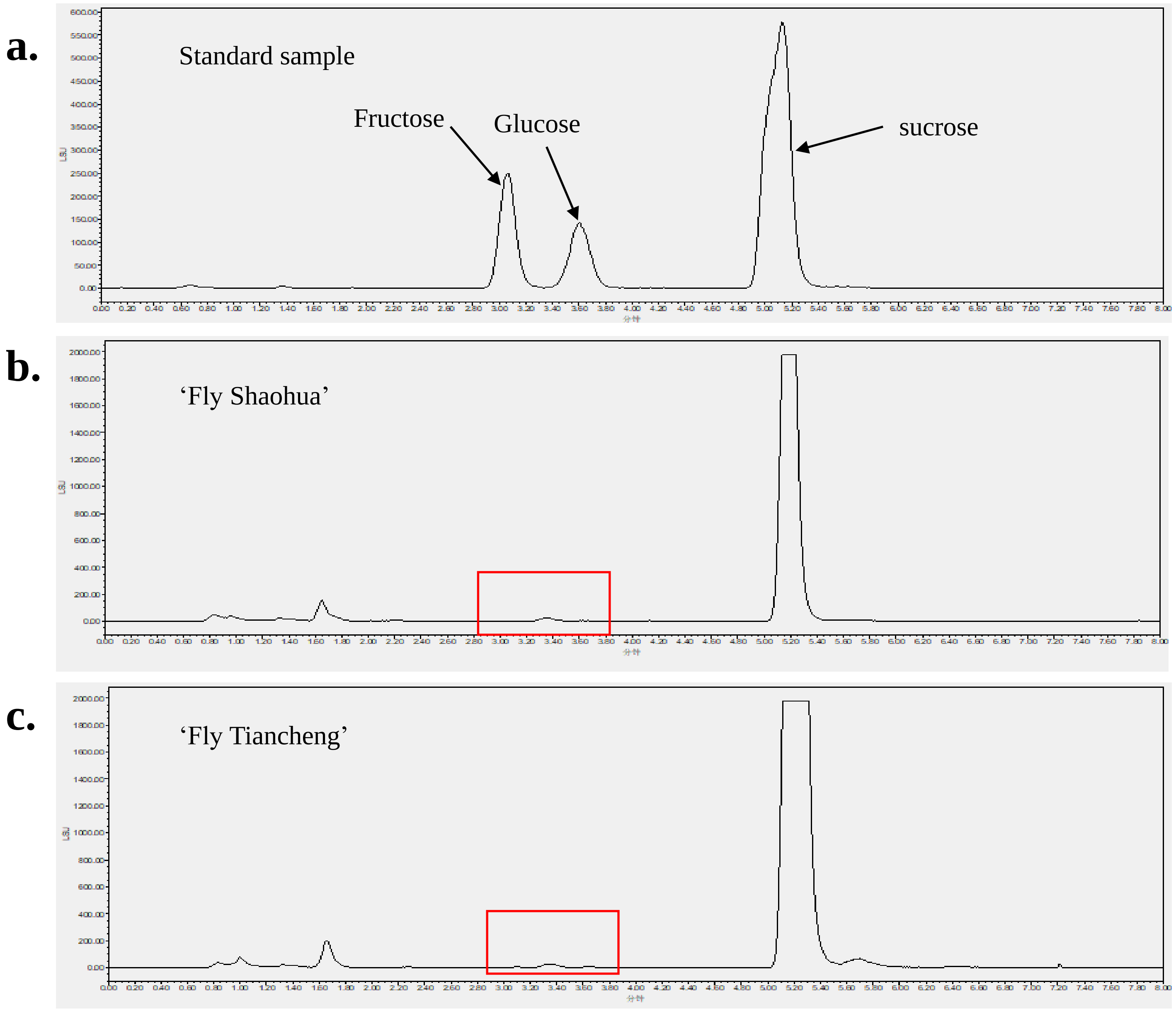

Figure S1. Chromatographic peaks of standard sample, ‘Fly Shaohua' and ‘Fly Tiancheng’ bulbs extracting solution. **a**, **b** and **c** represent the standard sample, ‘Fly Shaohua’ bulbs extracting solution and ‘Fly Tiancheng’ bulbs extracting solution, respectively. The red box shows the extremely weak chromatographic peaks of fructose and glucose.
